# Pregnancy and birth after intracytoplasmic sperm injection with normal testicular spermatozoa in a patient with azoospermia and tail stump epididymal sperm

**DOI:** 10.1590/S1677-5538.IBJU.2015.0296

**Published:** 2015

**Authors:** Betina B. Povlsen, Lin Da Aw, Rita J. Laursen, Sandro C. Esteves, Peter Humaidan

**Affiliations:** 1 Fertility Clinic, Skive Regional Hospital, Denmark; 2 Androfert, Andrology & Human Reproduction Clinic, Referral Center for Male Reproduction, Campinas, Brazil; 3 Faculty of Health, Aarhus University, Denmark

**Keywords:** Sperm Injections, Intracytoplasmic, Sperm Retrieval, Sperm Tail

## Abstract

**Main findings:**

An intriguing yet perplexing case report of a successful pregnancy and live birth with intracytoplasmic sperm injection using normal testicular sperm, after the finding of azoospermia in the semen analysis and discovering only tail stump abnormal sperm in the epididymis.

**Case hypothesis:**

A tail stump sperm defect of genetic origin was suspected. However, after obtaining normal testicular sperm we concluded that obstructive azoospermia, either idiopathic or secondary to multiple minor genital trauma was the plausible scenario. This has rendered the search of previous reports on a similar condition, but none was found. However, it has raised scientific thoughts for future research.

**Promising future implications:**

The importance of reporting this case is to alert urologists performing sperm retrieval that healthy and morphologically normal sperm may be found in the testis of azoospermic men with 100% tail stump epididymal sperm. Retrieval of normal testicular sperm obviates the need of a more complex investigation, including sperm electron microscopy. It also offers the possibility of utilizing such gametes for sperm injections rather than abnormal tail stump sperm that may be associated with a poor reproductive outcome.

## INTRODUCTION

As many as 186 million people are estimated to be affected by infertility worldwide, of which the male factor accounts for more than 50% of all cases of childlessness (1). While the introduction of intracytoplasmic sperm injection (ICSI) has undoubtedly offered opportunities to treat the most severe cases of male infertility, the advancement and modernization of assisted reproductive technologies (ART), however, still fail to answer the etiology of some rare and enigmatic conditions, as described in this case report, gearing to future research.

### 

#### Scenario

A 30-year-old male and his spouse were seeking treatment for their infertility condition at The Fertility Clinic Skive Regional Hospital. The couple presented with an infertility history of one year duration and no previous treatments. The male partner had undergone multiple semen analyses, all of which revealed azoospermia. The patient`s sexual, medical/surgical and family histories were essentially unremarkable. Notably, the patient was inquired about and confirmed that he had not had any history of sexually transmitted diseases or exposure to gonadotoxins. Childhood and pubertal development were also normal, but for the fact that he reported being a former goalkeeper playing handball with many hits on his genitals, which were mostly minor injuries with no need for medical assistance. He was a non-smoker with a normal body mass index, but reported alcohol consumption to a maximum of 2–4 units per day. His physical examination findings were unremarkable. The genitalia examination revealed a normal penis and both testes located in the scrotum, measuring approximately 20mL each in volume with no hydrocele. The epididymis were normal in size and consistency. Examination of the spermatic cord revealed a palpable normal vasa deferentia and absence of varicocele.

Semen analyses were repeated, confirming azoospermia after centrifugation of the liquefied specimens. Further investigations revealed a normal hormonal profile: FSH level of 1.5IE/L (normal range: 1.2–15.8IE/L), LH level of 4.4IE/L (normal range: 1.7–8.6IE/L), testosterone level of 17.87nmol/L (normal range: 11–34nmol/L) and prolactin of 274MIE/L (normal range: 90–580MIE/L). The chromosomal analysis showed a normal karyotyping with no Y chromosome deletion or cystic fibrosis mutations found.

**Figure 1:**
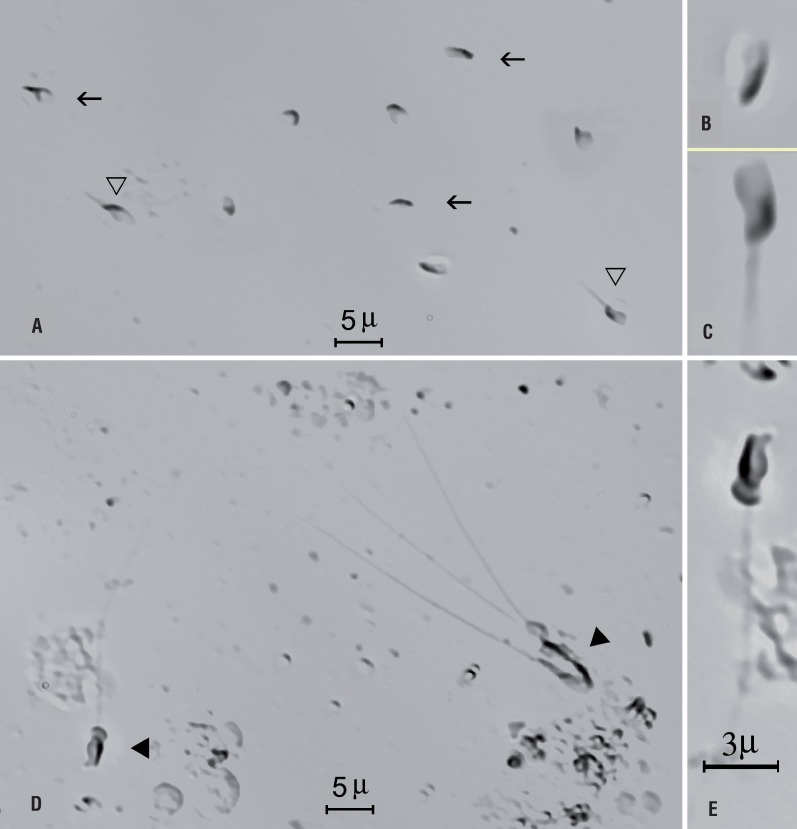
Photomicrographs of wet preparations containing spermatozoa obtained by percutaneous epididymal sperm aspiration (PESA; a-c) and testicular sperm aspiration (TESA; d, e) from an infertile 30 year-old male with azoospermia. Open black arrows and triangles indicate tail stump and no tail epididymal spermatozoa (a). In ‘b’ and ‘c’, individual spermatozoa with no tail (b) and tail stump (c) are highlighted. Black triangles indicate normal spermatozoa retrieved from the testicle (d). A spermatozoon with a fully developed tail is highlighted (e). photographs obtained using a Nikon Eclipse inverted microscope with Hoffman phase contrast optics (Nikon, Japan). The magnification bar shown in ‘e’ also applies for ‘b’ and ‘c’.

A diagnostic percutaneous epididymal sperm aspiration (PESA) was carried out in the epididymis head, which found all spermatozoa to be morphologically abnormal, even after repeating the procedure at the contra-lateral side. Of note, all retrieved spermatozoa were immotile and showed the same tail defect, namely either ‘tail stump’ or no tail (Figures 1 a-c). Sperm viability testing was not performed.

With no normal spermatozoa yielded on PESA, a diagnostic testicular sperm aspiration (TESA) was performed at the same operative time. Surprisingly, normal spermatozoa were obtained (Figures 1d and 1e). A careful microscopic examination revealed no tail stump spermatozoa in the TESA sample, of which approximately 10% retrieved spermatozoa exhibited sluggish non-progressive motility. Both PESA and TESA specimens were frozen, using the liquid nitrogen vapor technique, and stored. Subsequently, the couple underwent an ICSI cycle. The mature retrieved oocytes were micro-inseminated with TESA-normal and motile spermatozoa from the male partner. The couple successfully conceived and delivered a healthy baby at term. Two years later, the couple came back for replacement of the remaining embryos that had been stored, however, the attempt was unsuccessful. Recently, they donated the straws of frozen tail stump spermatozoa obtained from PESA and the normal spermatozoa from TESA for research purpose.

#### Case hypothesis and rational

Our initial hypothesis was “tail stump sperm defect of genetic origin” given the observation of 100% abnormal spermatozoa on PESA. However, after obtaining normal testicular sperm on TESA, of which about 10% were motile, we hypothesized that the patient had obstructive azoospermia either idiopathic or secondary to multiple minor genital trauma. Although viability studies would add to our report, we have not carried out viability testing in the PESA and TESA specimens. Given all spermatozoa retrieved from both epididymis were immotile and exhibited tail stump, our approach was to perform TESA in the same operative time. We retrieved sperm with normal tails by TESA, of which approximately 10% exhibited sluggish non-progressive motility, thus confirming viability in a proportion of the retrieved testicular spermatozoa. The importance of reporting this case is to alert urologists performing sperm retrieval that normal sperm may be found in the testis of men with tail stump epididymal sperm. The finding of normal testicular sperm by TESA will obviate the need of a more complex investigation including sperm electron microscopy. Also, it offers the possibility of utilizing such normal gametes for sperm injections rather than the abnormal tail stump sperm that may be associated with a poor reproductive outcome.

## DISCUSSION AND FUTURE PERSPECTIVES

The excitement of obtaining a successful pregnancy and healthy offspring by ICSI after an unexpected diagnosis of azoospermia and 100% tail stump epididymal spermatozoa, and the subsequent finding of normal testicular sperm, was the driving force of reporting this intriguing case.

Although anecdotally pregnancies have been reported in humans with the utilization of tail stump spermatozoa and ICSI, the abnormally shaped sperm may negatively influence the reproductive outcome (2–4). Moreover, given that a genetic, but still unmapped defect has been postulated to be the cause of such tail defects, there is a concern of transmitting a genetic abnormality to the male offspring that will render them infertile (5–7). As a matter of fact, such a genetic linkage, either direct or indirect, has been associated with a number of sperm defects in different species (5, 8).

In humans, the finding of 100% tail stump spermatozoa is rare (9). Although the origin of this defect is unknown, testes sections revealed that damage might occur during spermiogenesis in the latest stages of flagellum elongation at the spermatid stage, which resulted in a generalized, blocked formation of the flagellum associated with an absence of axonemes and accessory fibers (9, 10). Electron microscopy studies revealed that the ‘stump’ category exhibits the sperm tail region organized as uniflagellate, and the extremely short axoneme can have a ‘9+2’ or ‘9+0’ arrangement generally with dynein arms, while the ‘short tail’ category has a biflagellate arrangement and a ‘9+0’ or ‘9+1’ axoneme almost devoid of dynein arms (5).

Although a genetic linkage has been discussed in previous reports involving tail stump sperm (2–7, 11), there may be other possible explanations. In an earlier study evaluating 247 men with severe asthenozoospermia, Chemes and colleagues reported that most men presented with nonspecific flagellar anomalies, which were random, secondary alterations that affected a variable number of spermatozoa in different samples (6). In such cases, there was no familial/genetic inheritance, and the flagellar anomalies were secondary to different andrological disorders. It has been thus suggested that this aforementioned type of flagellar anomaly be differentiated from the less common fibrous sheath dysplasia that is associated with genetic abnormalities or familial inheritance (6, 7).

In our reported case, azoospermia was found in multiple ejaculates after centrifugation. In such cases, a distinction should be made between obstructive and non-obstructive azoospermia. When normal genitalia (testes, epididymis and vasa deferentia) and endocrine profile are found in a normal virilized adult male, the finding of spermatozoa within the epididymis is highly indicative of obstructive azoospermia (12), as shown in our patient. Obstruction in the male reproductive system can be congenital or acquired. Acquired causes include vasectomy, infection and genital trauma (13, 14). The most common congenital form of obstructive azoospermia (OA) is congenital bilateral absence of vas deferens (CBAVD), which is linked to mutations in the cystic fibrosis transmembrane-conductance regulator (CFTR) gene (13). Our patient, however, neither had a clinical genitourinary infection or surgery, nor were the vasa deferentia absent on physical examination. Therefore, the possible explanation for his condition would be either genital trauma as noted in his medical history, or a congenital idiopathic obstruction.

The importance of investigating CFTR mutations in a case like ours relies on the fact that such mutations have been implicated in bilateral epididymal obstruction even in the presence of normal, bilateral palpable vasa (15), as many as 47% patients with idiopathic bilateral epididymal obstruction carry CFTR mutations (16, 17). The most common identified mutations were IVS8–5T, ΔF508, R117H and L206W, but none of them were found in our patient. Young syndrome is another rare disease primarily characterized by a bilateral epididymal obstruction with azoospermia. However, bronchiectasis and chronic sinusitis are common features in patients with this disorder, and epididymal sperm is normal and motile (15).

The reason why all spermatozoa retrieved from the epididymis of our patient had abnormal tails is unknown. However, given normal sperm were found within the testis it is presumptive that the morphological alterations occurred within the epididymis after sperm release from the seminiferous tubules. We therefore speculate that the presence of tail stump sperm in our case scenario might be due to prolonged epididymal stasis resulting in senescent sperm as sometimes seen in post-vasectomy vas and epididymal fluid aspirates (18–20). Such sperm tail defects may be associated with other concomitant epididymal pathologies, namely subclinical microbial infections and antisperm antibodies.

Lastly, the delivery of a healthy baby is the ultimate goal in assisted reproductive techniques. This has been achieved in our case with the micro-insemination of oocytes using normal sperm retrieved by TESA. The reproductive outcome of ICSI, using testicular sperm retrieved from men with OA has been reassuring, and TESA is associated with few complications (14, 21). The chances of achieving a live birth and the profile of neonates born after sperm injection with a so-called normal epididymal or testicular sperm do not seem to be related to the cause of obstruction. Moreover, ICSI outcomes are comparable using frozen-thawed or fresh sperm retrieved from men with OA (22).

Despite it being an uncommon condition, the finding of azoospermia associated with 100% abnormal tail stump epididymal sperm may pose many challenges to the treating physicians. We hope the case presented here can help urologists to provide an even better management of patients, and encourage further research to enlighten the origin of this enigmatic condition.

## CONCLUSIONS

Urologists performing sperm retrieval should be aware that healthy and morphologically normal sperm may be found in the testis of men with obstructive azoospermia and 100% tail stump epididymal sperm.
